# Dendrimers as Potential Therapeutic Tools in HIV Inhibition

**DOI:** 10.3390/molecules18077912

**Published:** 2013-07-05

**Authors:** Jianqing Peng, Zhenghong Wu, Xiaole Qi, Yi Chen, Xiangbo Li

**Affiliations:** Key Laboratory of Modern Chinese Medicines, China Pharmaceutical University, Nanjing 210009, China

**Keywords:** dendrimer, HIV, gp120, CD4, dendriplex

## Abstract

The present treatments for HIV transfection include chemical agents and gene therapies. Although many chemical drugs, peptides and genes have been developed for HIV inhibition, a variety of non-ignorable drawbacks limited the efficiency of these materials. In this review, we discuss the application of dendrimers as both therapeutic agents and non-viral vectors of chemical agents and genes for HIV treatment. On the one hand, dendrimers with functional end groups combine with the gp120 of HIV and CD4 molecule of host cell to suppress the attachment of HIV to the host cell. Some of the dendrimers are capable of intruding into the cell and interfere with the later stages of HIV replication as well. On the other hand, dendrimers are also able to transfer chemical drugs and genes into the host cells, which conspicuously increase the anti-HIV activity of these materials. Dendrimers as therapeutic tools provide a potential treatment for HIV infection.

## 1. Introduction

The 2012 UNAIDS World AIDS Day report shows that 34 million people are infected with human immunodeficiency virus (HIV), the agent responsible for AIDS, and 1.7 million people died because of HIV-related diseases [1]. Therefore, AIDS still remains the serious problem around the World and a large amount of research is focused on the development of chemical agents and vaccines for HIV inhibition. 

The life cycle of HIV comprises a number of steps: first, HIV attach to the host cell and fuses with the membrane; then, the viral RNA reverse transcripts to form DNA and integrates into the host cell’s DNA; next, mRNA is formed via transcription and protein expresses; finally, the new virus is assembled automatically. Each of the steps could be considered as the target for intervention [2]. Among all the steps, the targets that are the focus of most attention are the attachment of HIV to the host cell and the reverse transcription of HIV in the cell nucleus. A lot of polymers, chemical agents and gene therapies are widely used to block these two steps for HIV replication, yet most of the treatments are lacking in effectiveness.

Dendrimers are highly branched macromolecules with uniform and controllable size, monodispersity and high density of peripheral functional end groups constructed through the sequential addition of branching units from an initiator [3]. The methods by which the dendrimers are synthesized result in precise control of the dendrimer size, shape and modified end groups. Thus, the dendrimers have been designed with specific functional end groups to preferentially interact with the envelope protein of HIV and receptors on the host cells in order to suppress the combination between HIV and host cells and later stages of HIV replication. Among all the developed dendrimers, the gel preparation of SPL7031 for intra-virginal use has successfully completed phase-I clinical trials [4].

Besides, dendrimers with large amounts of peripheral groups and interior cavities are potential vectors for chemical drugs, peptides and genes for HIV inhibition. These materials are capable to either interacting with the peripheral groups or be encapsulated into the cavities of dendrimers with hydrogen bonds, electrostatic and hydrophobic interactions [5,6]. Dendrimers would increase the stability of chemical drugs, and promote cellular uptake via functional end groups. For gene therapies, dendrimers can take the place of virus to transfer the interference genes into the target cells to suppress the replication HIV.

**Figure 1:**
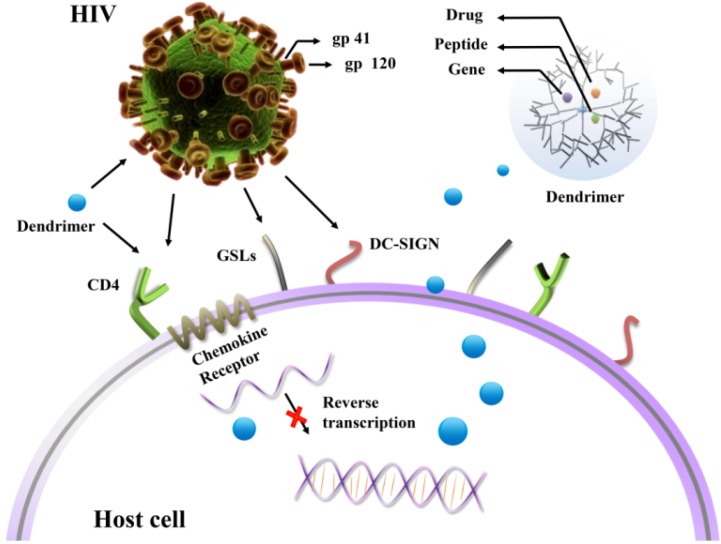
Dendrimers as potential agents and non-viral vectors of drug/gene delivery system for HIV inhibition.

The aim of this review is to report the role of dendrimers as both therapeutic agents and vectors of therapeutic materials (Figure 1). The application of various functionalized dendrimers as anti-HIV agents are classified according to different stages of HIV inhibition, while the studies on dendrimers as carriers are organized by the category of anti-HIV agents used. This review summarizes the progress achieved in the field of dendrimers as potential therapeutic tools for HIV inhibition highlighting that further studies are required for the development of more effective dendrimer-based therapies.

## 2. Functionalized Dendrimers as Potential Anti-HIV Agents

Functionalized dendrimers act as anti-HIV agents in two ways: first, to prevent binding of HIV to the host cells, which includes three potential binding sites CD4 receptors, glycosphingolipids (GSLs) and dendritic-cell-specific ICAM-3-grabbingnon integrin (DC-SIGN) receptors; second, to prevent replication of HIV in the host cells which includes reverse transcription of RNA and integration into the DNA of host cell (Table 1).

**Table 1:** The dendrimers with various functional groups for HIV inhibition.

Code name and reference	Dendrimer classification	Functional end groups	Combination sites	Stage of inhibition
BRI2932 (SPL2923) [7]	PAMAM G4	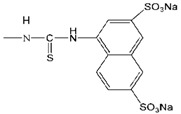	gp120	attachment and replication
BRI6195 (SPL6195) [7]	PAMAM G4	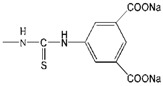	gp120	attachment
SPL7013 [8,9]	Polylysine dendrimer	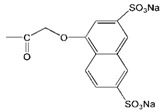	gp120	attachmentand replication
2G-S16 [10,11]	Carbosilane Dendrimer	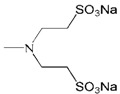	gp120/CD4	attachment
2G-C16 [11]	Carbosilane Dendrimer	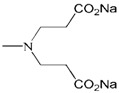	N/A	N/A
PS Gal 64mer [12]	Polypropylenimine tetrahexaconta- amine dendrimer	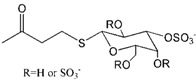	gp120	attachment
PLDG3 and SCSLD3 [13,14]	Polylysine and amphiphilic lysine dendrimer	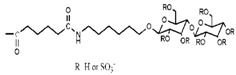	gp120	attachment
MVC-GBT [15]	Polypropylenimine dendrimer	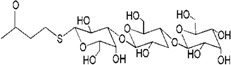	gp120	attachment
MVC-3SL [15]	Polypropylenimine dendrimer	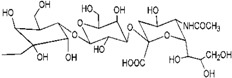	gp120	attachment
[G1]-CO_2_Na [16]	GATG dendrimer	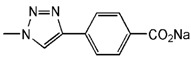	C-terminal domain of capsid	assembly
PAMAM [17,18]	PAMAM	-NH_2_	trans-acting responsive (TAR) RNA	replication

### 2.1. Prevention of HIV Attachment to the Surface of Host Cells

#### 2.1.1. CD4 Receptors

HIV infection starts with attachment of envelope protein gp120 to CD4 molecules on the host cell surface, followed by engagement of chemokine receptors (CXCR4 and CCR5) to form a CD4-gp120-chemokine receptor complex. Subsequently, envelope protein gp41 of the virus mediates the membrane fusion [19]. Thus, dendrimers with functionalized end groups were developed to combine with gp120 or CD4 molecules to impede the subsequent steps of HIV infection.

Ahead of the discovery of the mechanism of HIV inhibition, two polyanionic dendrimers BRI2932 (SPL2923) and BRI6195 (SPL6195) was found to inhibit the replication of HIV (strain III_B_) at an EC_50_ of 0.1 and 0.3 μg/mL respectively, with extremely low cytotoxicity to the host cells. A gp120 binding assay and virus adsorption assay indicated that both compounds have an effect on the docking of HIV to the host cells. Besides, SPL2923 at higher concentration (500–2,500 times of its EC_50_) could also block the later stages of HIV infection. Correspondingly, results from cellular uptake experiments testified that SPL2923 was capable of invading the host cell, whereas SPL6195 was not [7]. 

On the basis of the structure of SPL2923, three dendrimers-generation 3: polylysine, poly(amido amine) (PAMAM) and poly(propyleneimine) (PPI) with functionalized groups were synthesized for further development of a mature macrobicide concerning more its anti-HIV activities. Although the biological activity of the three modified dendrimers was essentially the same, the scale-up manufacture of the dendrimers and the ultimate formulation showed evident distinctions. The residual cobalt in the synthesis of PPI dendrimer and the risk of reverse Michael addition action of PAMAM made them not the optimum candidates for further development [20], thus the l-lysine-based polylysine dendrimer SPL7013 was elected as the optimum candidate for further studies of physicochemical properties and preclinical researches [21,22]. The nonhuman primate efficacy experiments showed that 6/6, 5/6, and 2/6 pig-tailed macaques intra-vaginally treated with the 5%, 3% and 1% w/w SPL7013 gels, respectively, were rid of SHIV (chimeric simian/HIV-1 virus) infection, which indicated a dose-dependent resistance to SHIV infection of SPL7013 [4]. Thus, the clinical product of SPL7013 was applied as gel preparation which contained 3–5% w/w SPL7013, and named VivaGel^®^. 

Further studies showed that the ability of SPL7013 to suppress HIV infection could be ascribed to the combination between the dendrimer and the V3 loop of gp120 [8,23]. It was not difficult to find that gp120 could be coated by SPL7013 through the electrostatic surface simulation [8]. Whereas, as chemokine receptors were engaged in the infection, the activity of SPL7013 were found closely related to the type of HIV strain involved, including CXCR4-using (X4) and CCR5-using (R5) strains. The discriminating activities on various HIV strains indicated that SPL7013 had conspicuous virucidal activity against X4 HIV-1 strains, but less activity against HIV-1 strains solely used CCR5 receptor [9]. Therefore, it was reasonable to conclude that the anti-HIV activity of SPL7013 was determined by the type of HIV strains as well.

**Figure 2:**
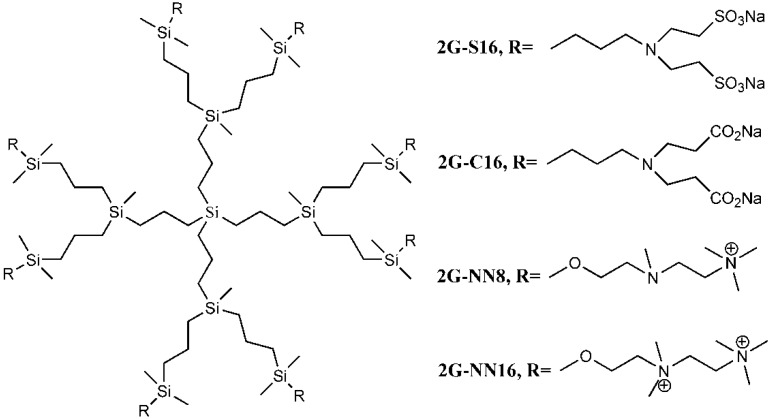
Generation 2 carbosilane dendrimers with different end groups.

Although the clinical safety [23,24] and antiviral activity [9,25] of SPL7013 had been verified, the lack of broad spectrum anti-HIV activity was the main defect of SPL7013 [9]. A new water soluble anionic carbosilane dendrimer with sulfonate end-groups (2G-S16) has been synthesized aiming at achieving more effective and larger spectrum virucidal activity against HIV (Figure 2).

As HIV is transmitted during sexual intercourse, 2G-S16 was designed to be administered vaginally. Two vaginal epithelial cell lines HEC-1A and VK2/E6E7 were used for anti-HIV activity evaluation. The results showed that 2G-S16 had a prominent anti-HIV activity *in vitro*. On the one hand, 2G-S16 could quickly inhibit HIV infection after eas short as 0.5 h via pre-treatment; on the other hand, only the concentration of 2G-S16 was above 5 μM for HEC-1A cells and 1 μM for VK2/E6E7 cells, respectively, that the virus could be decreased prominently. The ability of 2G-S16 to protect tight monolayers formed by epithelial cells from damage by HIV was verified in both the X4 and R5 HIV strains. In addition, 2G-S16 was demonstrated to impede HIV infection in peripheral blood mononuclear cells (PBMC) after transmission through monolayers. The mechanism of anti-HIV activity of 2G-S16 was assumed to be based on the binding to CD4 molecules or gp120. Thus, three dimensional computer models of 2G-S16, CD4 and gp120 were simulated and the binding simulation indicated that 2G-S16/gp120 showed higher affinity than 2G-S16/CD4 [10].

Besides, a transition metal-copper was incorporated into carbosilane dendrimers decorated with anionic carboxylate (2G-C16) and sulfonate moieties (2G-S16) to achieve neutral and anionic copper complexes (Figure 2). The cytotoxicity of neutral and anionic copper complexes was studied on HEC-1A and PBMC cells, indicating no toxicity up to 50 μM. With pre-treatment by the dendrimers, both 2G-C16 and 2G-S16 copper complexes showed higher inhibitory effects than the blank carbosilane dendrimers on HEC-1A and PBMC cells. The post-treatment on PBMC by copper complexes showed prominent anti-HIV activity as well, which was both dendrimer concentration and copper amount dependent [11]. The reason for the enhancement of HIV inhibition effect in the presence of copper was not determined in this study, which was unable to confirm whether the metal ion increased the combination between dendrimer and gp120/CD4 molecules or the complexes had an effect on the later stages of HIV replication.

#### 2.1.2. GSLs

In non-CD4-expressing cells the potential binding receptors of HIV have been supposed to be GSLs [26,27]. It has been verified by several studies that the gp120 of HIV virus would bind with the GSLs for cell entrance [28]. Thus, the dendrimer-based antagonists for HIV could be modified with carbohydrates moieties to mimic the local spatial structure of GSLs.

Galactose [12], cellobitose [13,14], globotriose and 3’-sialyllactose [15] have been employed as functional dendrimer end groups to suppress attachment of HIV to host cells. In accordance with the previous compounds, both of the galactose and cellobitose were sulfated to achieve effective inhibition activity.

The preferential interactions between specific sulfate and V3 loop of gp120 was verified by Richard and colleagues [22] through implementation of the inhibition of gp120 adherence to immobilized sulfatide by galactosyl ceramide (GalCer), sulfatide galactosyl ceramide (SGalCer), ceramide, dextran sulfate (DxS), and chondroitin sulfate (ChS). The previous studies showed that GalCer and its 3-sulfated derivative-SGalCer had high affinity to gp120 which mediated the HIV-1 infection, and a series of analogs of GalCer-modified dendrimers showed impressive anti-HIV activity [29,30]. However, the results showed that the best ligand for gp120 was SGalCer. Therefore, the randomly sulfated galactose moieties (including sulfate at position C-3) were modified on a poly- propylenimine tetrahexacontaamine dendrimer (PS Gal 64mer). The dendrimer with specific position C-3 sulfate modified galactose, akin to SGalCer, was certified by the HIV inhibition assays to be the most ideal HIV inhibitor candidate [12]. 

Compared with the position-specifically sulfated galactose modified dendrimer, randomly sulfated cellobitose was connected to a generation 3 polylysine dendrimer (PLDG3). PLDG with average 1.85 sulfate groups per glucose unit had very low cytotoxicity (CC_50_ > 1,000 μg/mL). The anti-HIV activity assay demonstrated that PLDG3 had less antiviral activity than dextran sulfate, curdlan sulfate and azidothymidine (AZT), but similar activity to dideoxycytidine (ddC). As low molecular weight oligosaccharides (such as cellobitose) have little or no anti-HIV activity [31], the high biological activity of PLDG3 was ascribed to the cluster effects of sulfated cellobitose on PLDG3 [13].

In addition, the sulfated cellobitose functional group was also attached to an amphiphilic lysine dendrimer (SCSLD3), which was covalently bound to a stearylamide. The long stearyl chain was reported to interact with the HIV lipid bilayer and destroy it, similarly to the function of surface active reagents with both hydrophilic (sulfated cellobitose) and hydrophobic ends (stearyl chain) [32]. The preliminary anti-HIV activity of SCSLD3 was determined to have as high activity as PLDG3 [14].

It is supposed that the specifically sulfated position of galactose on PS Gal 64mer is the key structure component for anti-HIV activity, while the specific-position sulfation is obviously not required by PLDG3 and SCSLD3. The cluster effect and compact structure of cellobitose on PLDG3 and SCSLD3 have been speculated to play important roles in the high biological activities [14].

Moreover, the carbohydrate portions of globotriosyl ceramide (Gb_3_) and hematoside (GM_3_)—globotriose and 3′-sialyllactose were covalently attached to polypropylenimine dendrimer to prepare multivalent carbohydrates (MVC-GBT and MVC-3SL). The effects of MVCs on HIV-1 infection had been tested on transformed T cells, primary PBMC, and the epithelial HeLa cell line-derived TZM-bl reporter cells. The results showed that both of the MVCs strongly inhibited infection of CXCR4 and CCR5-utilizing HIV-1 strains on T cells. In the determination of HIV inhibition on PBMC, the effects of endotoxin that would stimulate chemokines to inhibit infection was excluded and the activity was attributed to MVCs barely. The results showed that more than 75% cell fusion was inhibited by MVCs. As Gb_3_ and GM_3_ were reported to be involved in envelope protein mediated cell fusion [33,34,35], both of the MVCs were supposed to prevent the HIV infection through interference of combination between gp120 and cell surface [15].

#### 2.1.3. DC-SIGN Receptors

DC-SIGN receptor is a tetrameric C-type lectin mainly presented on the surface of immature dendritic cells (DCs) for modulating the immune response against pathogen [33,34]. In previous studies, DC-SIGN was testified as the receptors of a number of pathogens, including Ebola, simian immuno-deficiency virus (SIV), HIV and cytomegalovirus. The mechanism of DC-SIGN to act as a receptor had been exploited with high affinity for mannose glycans moieties on the pathogens [36,37,38]. For instance, HIV possesses a high density of the Man-α1,2-Man motif on the gp120 [39]. 

Thus, a nucleic acid-encoded carbohydrate library that focused on mannose and its derivatives suggested that an assembly of mannose with an aryl group at the 6-position of the terminal unit is beneficial for DC-SIGN binding. The binding affinity of the assemblies to DC-SIGN was measured and the assembly with highest affinity was covalently bound to PAMAM. The studies on the assembly modified PAMAM showed intensive inhibition on the interaction between gp120 and DCs at 10 μM (60 μM in mannose), whereas a natural polymer of mannose-mannan only partially inhibited the interaction at 500 μM in mannose [40].

### 2.2. Prevention of Replication of HIV in Host Cells

#### 2.2.1. Polyanionic Dendrimers

In addition to binding with the host cells, nanoscale size dendrimers are capable of getting into the host cells and blocking the viral life circle as well. Polyanionic dendrimers are the most studied HIV viral life circle microbicidesr. The first developed compound SPL2923 has been found to have an effect on the reverse transcription process at a concentration of 100 μg mL^−1^ (5-fold of the concentration used for inhibiting the attachment to host cells). However, SPL 6195 could not penetrate into the host cells and interfere with the later stages of HIV infection, even if the concentration of the compound was increased 5-fold. It was reasonable to conclude that the differences on the core structure and peripheral groups of dendrimers definitely affected their capability to restrain the replication of HIV [7]. 

Therefore, the similarities on peripheral groups between SPL7013/SPL7015 and SPL2923 implied that SPL7013/SPL7015 might have potential activity by inhibiting not only HIV entry but also HIV reverse transcription. The mechanism of HIV-1 inhibition by SPL7013 and SPL7115 was studied, and the results suggested that SPL7013 was capable of inhibiting the activity of reverse transcriptase (RT) of HIV-1 while SPL7015 was less active when incubated with HIV-1 RT in the medium without cells. However, both SPL7013 and SPL7115 failed to inhibit HIV-1 reverse transcription in HIV infected cells. The ineffectiveness of dendrimers was attributed to a lack of access to the cells or to the reverse transcription complexes in cells [8].

Besides, assembly of the HIV capsid protein is also a critical stage of HIV life circle, which has been developed as a target for anti-HIV drugs. A gallic acid-triethylene glycol (GATG) dendrimer was developed to inhibit the dimerization of the capsid (CA). The C-terminal domain of CA (CTD) was engaged in the formation of CA hexamers. Thus, the ability of GATG to inhibit the dimerization of CTD was tested first. The generation 1 (G1) GATG dendrimers were demonstrated via fluorescence and far-UV measurements to be able to interact with the dimeric CTD, especially [G1]-CO_2_Na and [G1]-OSO_3_Na, while generation 2 dendrimers failed to do so. In the determination of the binding sites on CTD, the absence of changes in chemical shifts and a large signal broadening in the HSQC-NMR spectra of dendrimers and monomeric CTD mixture indicated that both [G1]-CO_2_Na and [G1]-OSO_3_Na were able to bind to the residues of the dimerization helix of CTD, but not capable of changing the quaternary structure. Further, [G1]-CO_2_Na showed significantly activity in hampering the assembly of HIV-1 capsid *in vitro* [16].

#### 2.2.2. Polycationic Dendrimers

In addition to all of the polyanionic dendrimers mentioned above, unmodified polycationic dendrimers-PAMAM have been developed to inhibit binding of Tat protein to trans-acting responsive element (TAR) RNA of HIV [17,18]. The previous studies showed that the binding of Tat protein, a potent trans-activator of viral gene expression [41], to TAR RNA was crucial for the replication of HIV [42]. A lot of cationic molecules have been employed as HIV inhibitors, including polyamine acridine- based compounds [41] and polyallylamine hydrochloride (PAH) [43], which interacted with TAR RNA through electrostatic forces. Therefore, PAMAM possessing a large number of NH_2_ peripheral groups was conjectured to form stable electrostatic interactions with the phosphate groups of RNA. This hypothesis was verified by atomic force microscopy (AFM) and microgravimetric quartz crystal microbalance (QCM) analysis. The results showed that PAMAM generation 3 (G3) had a stronger affinity for TAR RNA than Tat protein, and the stable PAMAM/TAR complexes would impede further combination between TAR and Tat protein due to structure transition of TAR [17].

Further researches on the combination between PAMAM and TAR RNA by QCM measurement showed that it was described by a Langmuir-type isotherm, which suggested a monolayer combination. 2–5 generation PAMAM dendrimers were investigated in this experiments, as a result, the combination coefficient [K_D_^-1^]_s_ calculated according to the Langmuir equation indicated the order of the possibility of combination: G3 > G4 > G5 >Tat > G2. In other words, the binding between Tat protein and TAR RNA was capable to be prevented by the 3–5 generations of PAMAM. Furthermore, the order of migration times obtained from the capillary electrophoretic (CE) experiments reconfirmed the QCM measurement results, which was G3-RNA >G5-RNA > G4-RNA > Tat-RNA > G2-RNA. In conclusion, the 3–5 generation of PAMAM had the potential to inhibit replication of HIV and G3 PAMAM was the best candidate for anti-HIV inhibitors [18].

In addition, a kind of polycationic “viologen”-based dendrimer with excellent activity against replication of HIV-1 was developed. The dendrimers inhibited HIV-1 replication in MT-4 cells while they showed no inhibitory activity in human PBMC. The distinct anti-HIV activities were in line with the expression of heparan sulfate in the host cells. Thus, the inhibition of HIV replication was ascribed to the block of interactions between virus and heparan sulfate on the cell surface. This assumption was testified by the detection of charge distance of dendrimer moieties and heparan sulfate disaccharide unit. Besides, the dendrimers with spheroidal structure exhibited higher inhibition activity than those with comb-branched structures [44]. 

## 3. Dendrimers Acting as Potential Vectors for Drugs and Genes

The widely used anti-HIV chemical drugs have shown broad treatment failure and adverse effects which are related to their low cytotoxicity and high plasma concentration of drugs. Dendrimers as an ideal carrier could effectively bring the anti-HIV drugs into HIV-infected cells and take the place of virus to transfer genes into nucleus of HIV-infected cells in order to effectively interfere with the replication of HIV.

### 3.1. Dendrimers as Carriers for Chemical Drugs

The chemical anti-HIV drugs widely used for inhibition of reverse transcription are divided into three categories: nucleoside analogue reverse transcriptase inhibitors (NRTI), non-nucleoside reverse transcriptase inhibitors (NNRTI) and protease inhibitors (PI). Efavirenz (EFV), lamivudine (3TC), zidovudine (AZT) and didanosine (ddI) are the most studied anti-HIV drugs due to their most favorable resistance profiles. Except for EFV, the others are all NRTIs.

As monocytes/macrophages (Mo/Mac) were supposed to be the reservoir of HIV and played an important role on dissemination of virus throughout the body, antiretroviral drugs were expected to specifically intrude the Mo/Mac [45]. Lectin receptors were reported to be presented on the surface of the Mo/Mac [46,47]. Thus, connected with saccharides was supposed to be the most efficient way for drugs to get into Mo/Mac via combination of saccharides and lectin receptors [48,49].

Jain and colleagues employed generation 5 PPI dendrimer as an antiretroviral drug carrier. In order to specifically intrude into Mo/Mac, t-Boc-glycine and mannose were conjugated to PPI in order to decrease cytotoxicity to normal cells and increase aggregation of drugs in Mo/Mac. The toxicity of PPI, t-Boc-glycine modified PPI (TPPI) and mannose conjugated PPI (MPPI) was evaluated by the determination of haemolytic activity and cytotoxicity to HepG2 and Mo/Mac cells. The results showed that only PPI exhibited negligible haemolytic activity and cytotoxicity. The conjugation moieties increased safety of the carrier due to masking the terminal amino groups which decreased the positive charges. In addition, cellular uptake of EFV by Mo/Mac was found to be conspicuously increased through MPPI and TPPI encapsulation. Especially, the cellular uptake of MPPI/EFV is 12 times higher than that of free drug and 5.5 times higher than that of TPPI/EFV. Although both MPPI and TPPI achieved targeted delivery of EFV, the modified PPI carriers intruded into Mo\Mac in different way. MPPI got into the cells via combination with the lectin receptors presented on the surface of Mo/Mac, while TPPI were taken up through phagocytosis [50]. However, the anti-HIV activity of MPPI/EFV and TPPI/EFV had not been detected directly in this study.

The MPPI was also employed as carrier of 3TC-another antiretroviral drug. Anti-HIV activity of free 3TC, PPI/3TC and MPPI/3TC had been determined on the MT2 cell line through detecting p24 antigen on the surface of HIV by ELISA. The results showed that PPI/3TC and MPPI/3TC possessed higher anti-HIV activity than free 3TC at a concentration as low as 0.019 μM, while PPI exhibited significant cytotoxicity to MT2 cells which limited the application of PPI as a drug carrier. Further, the specific combination between mannose and Con A, a well-investigated lectin, was detected through *in vitro* agglutination assay. In accordance with previous studies, the results demonstrated that MPPI would specifically bind with lectin receptors. [51].

Triphosphorylated zidovudine (AZTTP) and triphosphorylated didanosine (ddITP) were also prepared as nano-formulations for efficient central nervous system targeting. Four nanocarriers have been studied in this research: PEG-PEI (NG1), Pluronic F68-PEI (NG2), PEG-PEI (NG3) and PAMAM-PEI-PEG (NG4). Besides, an ApoE peptide, targeting to the brain-specific apolipoprotein E receptor, was employed to combine with the carriers aiming at penetrating the blood-brain barrier (BBB). The results of cell accumulation showed that NG3 exhibited the fastest and highest capture by macrophages, followed by NG1, while NG2 and NG4 showed lower efficacy. In the antiviral efficacy determination, the macrophages were pre-treated with free AZTTP/ddITP and formulations before inoculation with HIV. The results showed that the nano-formulations displayed higher antiviral activity than free drugs, and the drugs encapsulated by NG4 and ApoE-NG1 displayed the highest antiviral efficacy. Hence, it was reasonable to conclude that the antiviral activity depended not only on the cellular uptake of formulations [52]. 

In addition to directly bringing drugs into targeted cells, dendrimers were designed as a steric cap for antiretroviral drugs as well. The antiretroviral drug and dendrimer were connected by HIV-1 protease cleavable linkers-specific sequence peptides. Thus, the steric caps would block the entrance of drugs into the proteasome in the absence of HIV-1 protease. The results showed that the entrance into the proteasome could be effectively blocked through binding with generation 3 lysine dendrimer. Therefore, the application of dendrimers with adequate size provided the foundation for the design of proteasome inhibitors to selectively kill HIV-infected cells [53].

### 3.2. Dendrimers as Carriers for Peptides

Dendrimers could be also employed as non-viral vectors of HIV-derived peptides for DCs based immunotherapy [54,55]. DCs played an important role in the development of immunotherapy against HIV infection due to their unique function in inducing innate immunities. However, DCs-based vaccines have shown low efficacy in clinical HIV trials, which was ascribed to the lack of persistence of antigen loaded in DCs [56]. Therefore, the HIV-derived peptides, employed as antigens, were encapsulated by a water soluble carbosilane dendrimer 2G-NN16 (Figure 2) in order to be captured by DCs for efficient immune responses.

Three peptides derived from Nef, Gp160 and P24 HIV proteins were mixed with 2G-NN16 to form peptide-dendrimer complexes [55]. The efficiency of the complexes for HIV immunotherapy was determined on DCs. The HIV-derived peptides showed enhanced uptake by immature and mature DCs (iDCs and mDCs) when formed complexes with 2G-NN16. As Gp160/2G-NN16 showed the earliest and strongest uptake by DCs, more characteristics of the complex was further tested in the following experiments. The uptake of 2G-NN16 and 2G-NN16/Gp160 was proved to have no significant effect on the iDCs phenotype, mDCs maturation ability and migration of DCs which was crucial for presenting antigens to T cells. And the complexes showed no impact on allogenic T helper cells stimulation via mDCs and changing of cytokines (IL-12p70 and TNF-α) *in vitro* as well. In a word, 2G-NN16 could be developed as antigen delivery carrier for DCs-based immunotherapy [54].

### 3.3. Dendrimers as Carriers for Genes

Generally, gene therapies for HIV inhibition can be divided into two categories: (1) oligodeoxynucleotides (ODNs) involving the HIV polypurine tract element mRNA (PPT), HIV anti-transactivation responsive gene (TAR), HIV gene expression modulator 91 (GEM 91), HIV replication mRNA (REV) and a random mix of two of the ODNs to form a cocktail; (2) siRNA involving three sequence: *siP24*, *siGAG1*, *siNEF* and cocktails. The genes were initially transferred into cells via viral vectors. However, the use of viral vectors suffered from several defects. On the one hand, the viral vectors would induce severe immune reactions. On the other hand, the genes would bind non-specifically to serum proteins which decreased the bioactivity and induced toxic effect [57]. Thus, a new non-viral vector was required to play the role of gene carriers.

The most studied non-viral carriers for ODNs and siRNA are generation 2 carbosilane dendrimers, including 2G-NN8 and 2G-NN16 (Figure 2). The carbosilane dendrimers have been shown to have very low cytotoxicity to PBMC, macrophages and DCs below 5 μM via (3-(4,5-dimethyl- thiazol-2-yl)-2,5-diphenyltetrazolium bromide (MTT) assay and lactate dehydrogenase (LDH) assay [58,59,60]. Furthermore, the researches demonstrated that ODNs [61] and siRNA [62] could form stable dendriplexes with dendrimers, and the dendriplexes were able to degrade in cells for further transfection. Besides, the size, zeta-potential and morphology of dendriplexes were determined by dynamic light scattering (DLS), atomic force microscopy (AFM) and transmission electron microscopy (TEM). Taking into account of all these studies, carbosilane dendrimers were regarded as ideal vectors for ODNs and siRNA.

#### 3.3.1. ODNs

Louis Chonco and colleagues were the first to employ 2G-NN8 and 2G-NN16 as carriers for ODNs to form dendriplexes. The studies showed that the dendriplexes were able to protect ODNs from binding to both bovine serum albumin (BSA) and human serum albumin (HSA). The dendriplex formed by 2G-NN16 and TAR was shown by confocal microscopy to be internalized into the PMBC, and the ODNs alone showed non-sequence specific inhibition effects on PBMC and lymphocyte T-cells (MT-2), however, the dendriplexes exhibited sequence specificity in HIV inhibition to some extent [58].

Further, the dendriplexes formed by 2G-NN16 and ODNs have been applied to some other cells. The transfection efficiency of dendriplexes was determined by confocal microscopy and flow cytometry on PBMC, DCs, lymphocytes (SupT1), astroglia (U87MG), neuroblastoma (SK-N-MC) and transformed trophoblastic (JEG-3 and JAR) cell lines. The results showed that the dendriplexes were capable of entering into the PBMC and exhibit significant cellular uptake in the case of the last four cell lines (above 90%). Besides, HIV-1 inhibition assays implemented on MT-2 and PBMC demonstrated that the dendriplexes formed by cocktails (2G-NN16/TAR/GEM 91 and 2G-NN16/TAR/REV) exhibited more than 60% inhibition rate [59]. 

Subsequently, the dendriplexes formed by 2G-NN8, 2G-NN16 and 2G-NN32 with triflate groups (CF_3_SO_3_) and ODNs were demonstrated to have higher transfection ability than ODNs alone through flow cytometry and confocal microscopy. However, the anti-HIV activity determined *in vitro* showed no effect on HIV inhibition. The low efficacy was ascribed to the strong combination between dendrimer and ODNs [60]. Therefore, the degradation ability of the dendriplexes would definitely affect the activity of ODNs.

#### 3.3.2. siRNA

As a carrier for siRNAs, 2G-NN16 provided a strong protection for siRNA challenged with heparin and ribonuclease (RNase). The transfection efficiency of dendriplexes was implemented on both Sup T1 cells and HIV-infected PBMC. Interestingly, naked siRNA showed successful uptake by Sup T1 cells, but was unable to enter the HIV-infected PMBC during the first 3 hours. The dendriplexes exhibited the highest transfection efficiency at a 2G-NN16/siRNA ratio of 1 or 2. When the 2G-NN16/siRNA ratio was more than 2, the higher +/− charge ratios would inhibit the viability of cells. Besides, naked siRNA showed sequence-specific and concentration-dependent activity against the housekeeping gene (GAPDH) in uninfected SupT1 cells and HIV replication in infected PMBC via electroporation. Further, the activity of dendriplexes was tested directly on cells. The results indicated that the dendriplexes possessed higher inhibition activity on GADPH in SupT1 cells and HIV-infected PBMC than naked siRNAs, especially with the cocktails of siRNA [63]. 

The dendriplexes formed by 2G-NN16 and siRNAs have been further applied for brain targeting. Transfection efficiency determination and transcytosis through an *in vitro* blood-brain barrier (BBB) model were implemented on astrocytoma cells (U87MG). Unexpectedly, the dendriplexes formed at a 2G-NN16/siRNA ratio of 8 exhibited the highest transfection efficiency, which was distinct from the previous study in which the lower ratio showed higher transfection efficiency. Both of the naked siRNAs and dendriplexes were demonstrated to successfully cross the monolayer barrier. The dendriplexes showed a dose-dependent HIV inhibition up to 85% on HIV infected U87MG cells [64].

Both of the researches above discussed the stability of dendriplexes to heparin. The results showed that the concentration of heparin would affect the release of siRNA from the dendriplexes. In addition, a study on the stability of dendriplexes formed by PPI and ODNs in the presence of heparin concluded that heparin at physiological concentration could destroy the dendriplexes formed by open shell glycodendrimers, while the dendriplexes based on unmodified PPI were stable [65].

Aside from carbosilane dendrimer, polycationic dendrimer PAMAM and phosphorus-containing dendrimer-G_4_ (NH^+^Et_2_Cl^−^)_96_ had been developed as carrier for gene therapies as well. The PAMAM and siRNAs, especially the cocktails, formed dendriplexes that not only exhibited conspicuous inhibition of HIV replication *in vitro*, but also effectively suppressed HIV infection in viremic RAG-hu mice. This was the first time that the dendriplexes were testified effectively for HIV inhibition *in vivo* [66]. In addition, the G_4_ (NH^+^Et_2_Cl^−^)_96_ dendrimer were demonstrated to be effective an vector for both GEM 91 and *siNEF*. The G_4_ (NH^+^Et_2_Cl^−^)_96_/*siNEF* dendriplex showed notably higher inhibition activity than siNEF alone on HIV infected MT-2 cells and PBMC [67]. 

## 4. Conclusions

We have reviewed the current status of dendrimer-based therapies for the treatment of HIV infection, and highlight the potential role of dendrimers as both therapeutic agents and non-viral vectors. As dendrimers have a lot of peripheric active groups, modified dendrimers have the ability to specifically combine with gp120 or CD4 molecule in order to block the attachment of HIV to the host cell. The functional groups involve the carboxylate groups, sulfonate groups and carbohydrate portions of GSLs. Especially, some of the dendrimers with sulfonate groups have been demonstrated to have an effect on the replication of HIV.

Besides, the application of dendrimers as carriers of chemical agents and genes is impressive. The dendrimers improve the efficiency of cellular uptake and anti-HIV activity on host cells, while the anti-HIV activity of the dendrimer-based formulations is not perfectly in accordance with the cellular uptake results. Hence, it’s reasonable to conclude that the release profile of drugs and the degradation ability of dendriplexes play an important role for anti-HIV activity as well.

In conclusion, dendrimers with specific functional groups offer significant benefits to prevent HIV infection. However, on the one hand, further investigation should concentrate on the development of large scale preparation of the functionalized dendrimers with high anti-HIV activity and safety. On the other hand, the dendrimer-based drugs and genes delivery systems require more studies on the *in vitro* and *in vivo* anti-HIV activity to obtain the optimum formulations, and the biodegradablity of dendriplexes which affects the activity of genes needs to be further investigated for more effective prevention of HIV infection.
